# Effect of Selenium-Enriched Garlic Oil against Cytotoxicity Induced by OX-LDL in Endothelial Cells

**DOI:** 10.1155/2014/537652

**Published:** 2014-05-29

**Authors:** Cheng Yang, Kai Cui, Yutao Diao, Min Du, Shumei Wang

**Affiliations:** ^1^School of Public Health, Shandong University, NO. 44 West Wenhua Road, Jinan 250001, China; ^2^School of Public Health Administration, Liaoning Medical University, Jinzhou 121000, China; ^3^Shandong Academy of Medical Sciences, Jinan 250012, China

## Abstract

*Objective*. To detect the effect of selenium-enriched garlic oil (Se-garlic oil) against cytotoxicity induced by ox-LDL in endothelial cells. *Methods*. Se-garlic oil was extracted by organic solvent extraction. High performance liquid chromatography (HPLC) was used to detect the content of allicin in the Se-garlic oil. Hydride generation atomic fluorescence spectrometry (HG-AFS) was used to detect the content of Se in the Se-garlic oil. ECV-304 cells were separated into five groups (blank, ox-LDL, and low-, medium-, and high-dose Se-garlic oil). Methyl thiazolyl tetrazolium (MTT) assay was used to detect the cytoactivity of each cell group after culturing for 24, 48, and 72 hours. Flow cytometry (FCM) stained with annexin V-FITC/PI was used to detect the apoptosis of the cells from the blank, Se-garlic oil, ox-LDL, and Se-garlic oil + ox-ldl groups after 48 hours of incubation. *Results*. The amount of allicin in Se-garlic oil was 142.66 mg/ml, while, in Se, it was 198 mg/kg. When ox-LDL was added to low-, medium-, and high-dose Se-garlic oil, the cell viability rates of ECV-304 cells treated in the three groups were all higher, while the apoptosis rates were significantly lower than those of the ox-LDL group (*P* < 0.05). However, there was no significant difference between the apoptosis rates of the blank, Se-garlic oil, and Se-garlic oil + ox-LDL groups (*P* > 0.05). *Conclusion*. Se-garlic oil could inhibit the cytotoxic effect induced by ox-LDL in endothelial cells.

## 1. Introduction

Oxidized low-density lipoprotein (ox-LDL) can cause injury and disability to vascular endothelial cells. This is considered to be related to the beginning of atherosclerosis (AS) formation [1, 2].

Research has found that garlic and its related products could counteract risk factors which would lead to AS [3]. Garlic not only could lower blood lipid level but also has an anticoagulant effect. Furthermore, garlic has an antioxidant effect in which it can cut off the oxidation of LDL, so it can prevent the formation and toxic effect of ox-LDL [3–6].

Selenium (Se) is one of the essential microelements, and it can lower endothelial cells' apoptosis and expression of adhesion molecules, which are caused by oxidative stress, high-fat diet, and other factors. Se is also known to be an anticoagulant and antioxidant [7–11].

Efforts have been made to combine garlic with Se to enhance the ability to counteract AS. Our research group, in a previous animal assay, found that allicin combined with Se can lower the blood lipid level of rabbits induced by a high-fat diet [12]. However, we found that garlic is opposite to Se in pH value in vitro, so their bioactivity is lowered, when they are simply mixed. Se-garlic is cultivated in selenium soil. Garlic can absorb inorganic Se, then changing it into organic Se by biological accumulation and biological transformation. In this way compared to inorganic Se, the hepatotoxicity and nephrotoxicity are less, and a moderate price and convenience can be attained.

Animal studies have found that Se-garlic has higher bioactivity than garlic or Se alone, and it is not significantly accumulated in the liver and kidneys [13, 14].

There has been little research on Se-garlic's effect against AS. The purpose of the present study was to find whether Se-garlic oil can act against the cytotoxicity induced by ox-LDL in endothelial cells.

## 2. Materials and Methods

### 2.1. Experimental Method 

#### 2.1.1. Preparation of Garlic Oil and Determination of Allicin and Selenium Content in the Oil

We extracted the garlic oil from the Se-enriched garlic (Shandong Xingfa Agricultural Science and Technology Co. LTD.) by an organic solvent extraction process. High performance liquid chromatography (HPLC) was used to detect the amount of allicin in the Se-garlic oil. Hydride generation atomic fluorescence spectrometry was used to detect the content of Se in the Se-garlic oil.

#### 2.1.2. Cell Culture and Treatment

The human umbilical vein endothelial cell line EVC-304 was cultured in RPMI1640 medium (Gibco, Los Angeles, USA) supplemented with 10% heat inactivated FBS, 100 *μ*g/mL penicillin, 100 *μ*g/mL streptomycin, and 2 mM L-glutamine and maintained in a humidified atmosphere with 5% CO_2_ at 37°C. The cells were subcultured twice each week and the exponentially growing cells were used in all treatments. Before treatment, cells were washed with PBS and fresh medium was added. At the time of treatment, working solutions were diluted accordingly in RPMI1640. The drugs were added to the cells 12 h after the subculture.

#### 2.1.3. Cell Viability Rate Assay

Cell viability rate was assessed by methyl thiazolyl tetrazolium (MTT) assay (Mosmann, 1983). The assay calculated the inhibition rate of cell viability treated by the drug. The design assay of the Se-garlic oil impact on the cell viability of EVC-304 cells included blank and seven-dose groups of the Se-garlic oil (5, 10, 20, 40, 80, 160, and 320 *μ*L/mL) and, for ox-LDL-induced cytotoxicity on EVC-304, four doses of concentrations of ox-LDL (10, 20, 40, and 80 *μ*g/mL) (Beijing Xiesheng NZCB). Each was done three times.

In the MTT assay, the untreated cells were generally in the exponential growth phase, and single-cell suspensions of 1 × 10^5^ cell/mL were inoculated into three 96-well plates, 200 uL per well. Cell groups were exposed to different concentrations of the drug for various time periods. Three plates were made and were cultivated for 24, 48, and 72 hours.

20 uL of MTT solution was added to each well 4 hours before the end and then centrifuged and the supernate was discarded. 150 uL of dimethyl sulfoxide (DMSO) solution was added into each well. Absorbance values (*A*) were detected by a microplate reader (mode FL 330, BioTek Instruments, Winooski, VT) at dual wavelength of 570/630 mm after shaking slightly for 10 minutes. The number of cells was also established using a hemocytometer, and all the results represented the average. The above experimental procedures were repeated three times. The viability rate of cells was calculated by using the following formula: viability of cell (%) = (the average value of the experimental group/the average value of the control group) × 100%.

#### 2.1.4. Testing the Se-Garlic Oil's Effect against the Cytotoxicity Induced by Ox-LDL in EVC-304 Cells

Based on 50% effective concentration of inhibition in the above assay, 40 *μ*g/mL ox-ldl was selected as the inducing cytotoxicity concentration in the test. The design assay of Se-garlic oil against the cytotoxicity induced by ox-LDL in the EVC-304 cells included five groups: blank, ox-LDL, and ox-LDL+ three dose concentrations of the Se-garlic oil (15, 30, and 60 *μ*L/mL). The MTT test was used to detect the cell activity rate. The experimental procedures were repeated three times.

#### 2.1.5. Cell Apoptosis Assay

The Se-enriched garlic oil's effects against apoptosis induced by ox-LDL were tested using a flow cytometer. The design assay included four groups: blank, ox-LDL (40 *μ*g/mL), Se-garlic oil (30 *μ*L/mL), and ox-LDL (40 *μ*g/mL) + Se-garlic oil (30 *μ*L/mL).

Forty-eight hours after culture, cells were trypsinized; 1 × 10^6^ cells per sample were washed twice with ice-cold PBS solution, and then 5 *μ*L of annexin V-FITC (AV) and 10 *μ*L of propidium iodide (PI) (Sigma Chemical Company, St. Louis, USA) were added to 100 *μ*L of cell suspension, followed by incubation for 15 min at room temperature in the dark. Finally, 400 *μ*L of binding buffer was added to each sample, and then the mixture was filtered by a 300-mesh nylon net before EPICS XL (Beckman Coulter, Bren, CA, USA) flow cytometry. EXPO32 ADC analysis software was used to analyze the data.

### 2.2. Statistical Analysis

SAS 9.1 was used for statistical analysis. Single factor analysis of variances was used for comparison among groups. The least-significant difference (LSD) test was performed for comparison between two groups. Multiway ANOVA was used for interaction. There was statistical significance if *P* value was below 0.05.

## 3. Result

### 3.1. Determination of Allicin and Selenium Content

**Figure 1:**
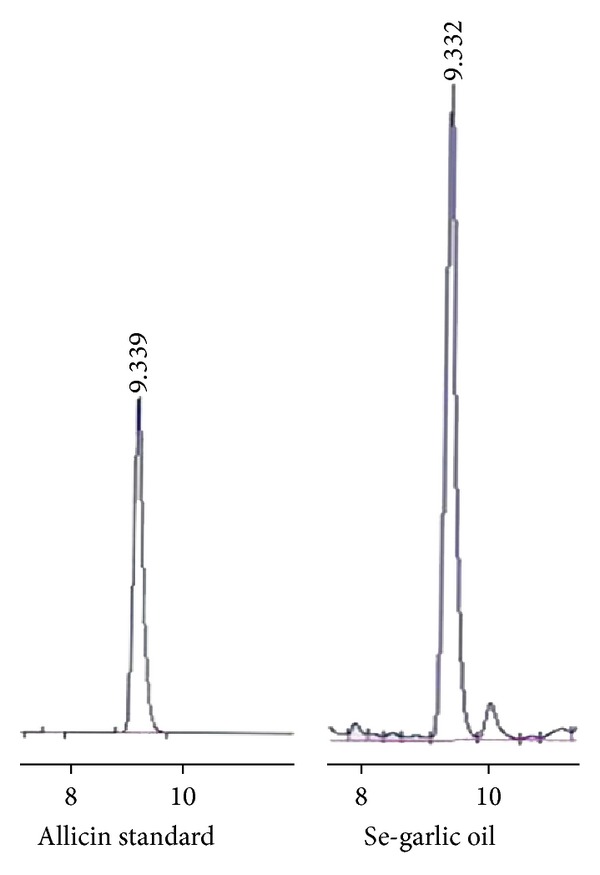
Content of allicin in raw Se-enriched garlic.

As shown in Figure 1, allicin's retention time was 9.339 min. A linear regression model was adapted to fit the data. The peak area was set as the dependent variable, and the content of allicin was set as the independent variable. The regression equation was *Y* = 13.086*X* + 509.977 (*R* ^2^ = 0.998, *P* < 0.01). The content of allicin in the Se-enriched garlic oil was calculated as 142.66 mg/mL, and the content of Se detected by hydride generation atomic fluorescence spectrometry was calculated as 198 mg/kg.

### 3.2. Cell Viability Rate Assay

**Figure 2:**
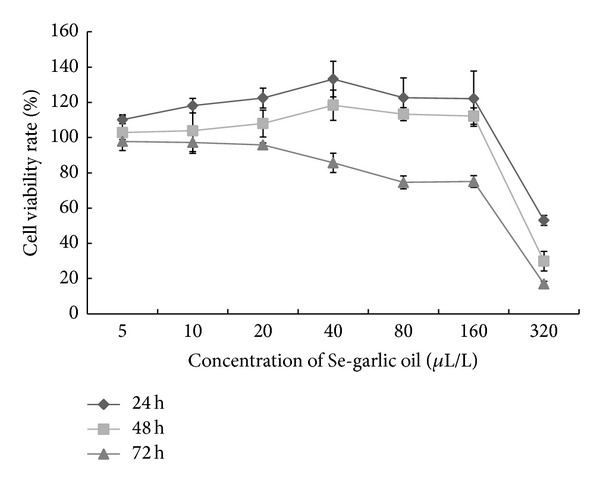
Curve of ECV-304 cell activity rate.

As shown in Figure 2, EVC-304 cells were treated with 0, 5, 10, 20, 40, 80, and 160 *μ*L/mL of Se-garlic oil using the MTT method. The cell proliferation rates showed time and dose dependence.

Based on EC4-304 cell's survival rate concentration-effect curve induced by the Se-garlic oil, we chose 30 *μ*L/L to be the middle concentration group to counteract ox-LDL in later experiments. We then respectively chose half time and 2 times (15 uL/L, 60 uL/L) as the low and high concentration groups.

**Figure 3:**
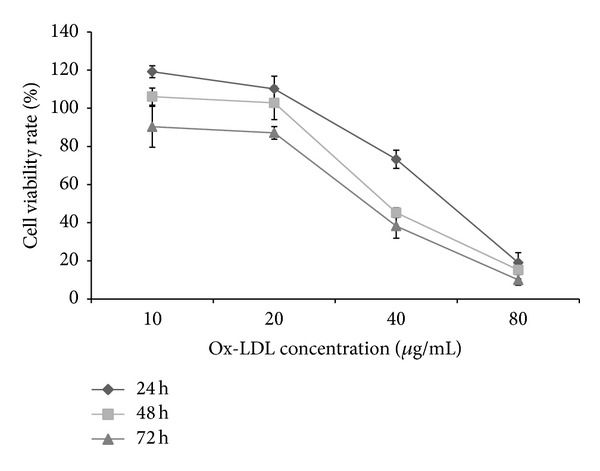
Curve of ECV-304 cell activity rate.

As shown in Figure 3. In order to find the effect of Se-garlic on cell injuries induced by ox-LDL, we chose the ox-LDL concentration which could inhibit cell activity in all three time groups with a cell activity rate near 50% (40 ug/mL) for the later experiment.

### 3.3. Se-Garlic Oil's Effect against Cytotoxicity Induced by Ox-LDL in EVC-304 Cells

**Figure 4:**
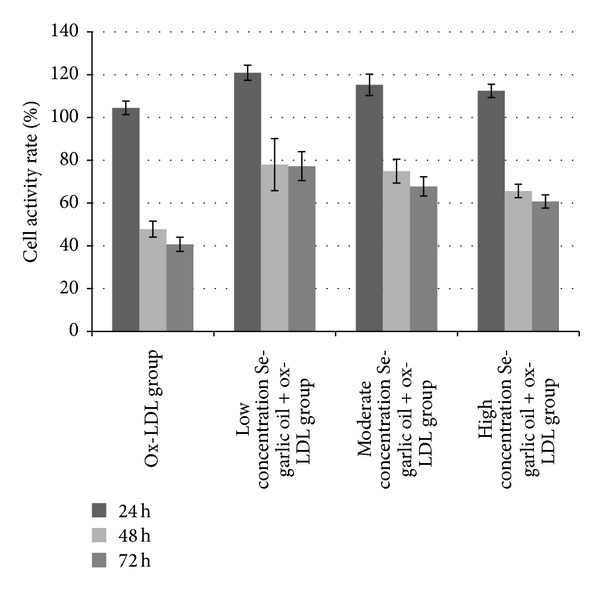
Effect of the Se-garlic oil on ECV-304 cell activity rate.

As shown in Figure 4, as results of one-way analysis of variance, the cell activity rates of low, medium, and high consistency groups were all higher than the ox-LDL group after 24, 48, or 72 hours (*P* < 0.05). When the interaction of time and concentration was eliminated by multivariate analysis of variance, there was no statistically significant difference among low, medium, and high consistency groups after 24 hours (*P* = 0.29). The cell activity rate of the high consistency group was lower than that of the low and medium consistency groups after 48 hours (*P* = 0.01), and there was no statistically significant difference between the low group and medium consistency group (*P* > 0.05). There was a statistically significant difference only between the low and high consistency groups after 72 hours (*P* = 0.02). At the same concentration, there was no statistically significant difference between cell activity rates after 48 and 72 hours (*P* > 0.05), both of which were lower than cell activity rates after 24 hours (*P* < 0.05).

### 3.4. Cell Apoptosis Assay

**Figure 5:**
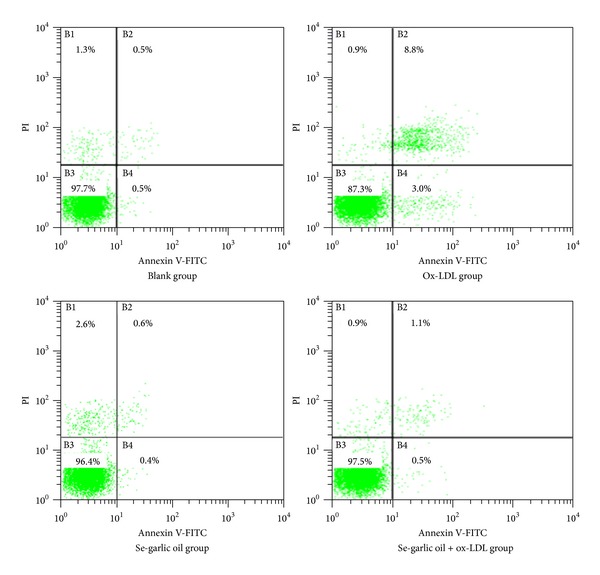
Apoptosis rate in different groups.

**Figure 6:**
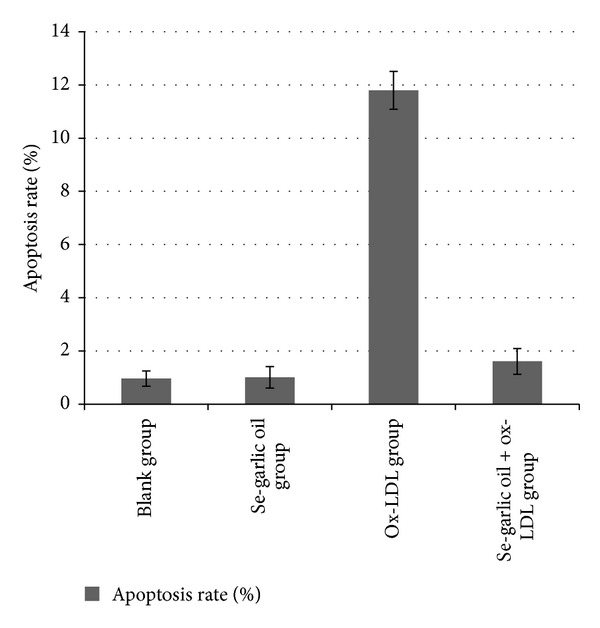
Comparison of apoptosis rate in different groups.

Results of the flow cytometry capacity model (FCM) are listed in Figures 5 and 6. The apoptosis rate of the ox-LDL group was significantly higher than the others (*P* < 0.05), and there was no statistically significant difference among the other groups (*P* > 0.05).

## 4. Discussion

Endothelial cells' survival rate decrease and apoptosis increase are the two main features of endothelium injuries. Garlic oil (the main component of Se-garlic) was extracted to study Se-garlic's effect on endothelium protection from these two aspects. Tests found that either allicin or selenium in Se-garlic is significantly higher than that of traditional garlic [15–18].

The cell activity rate curve affected by the Se-garlic oil showed that the cell activity rate decreased as the concentration of Se-garlic oil increased over time and that the cell activity rate first increased and then decreased after 24 and 48 hours. This trend suggested that both garlic and Se can stimulate cell activity rate at a low concentration or in a short time, but they will inhibit cell activity rate at a high concentration [19, 20].

According to the ox-LDL-induced curve, when the concentration of ox-LDL was at 10 or 20 *μ*g/mL or the time was 24 or 48 h, the cell activity rate approached 100%, while when the concentration was 40 *μ*g/mL, cell activity rate approached 70% after 24 h and 50% after 48 and 72 h. However, when the concentration was 80 *μ*g/mL, it had a significant effect on the cell activity rate, and the cell activity rate was below 20%. As a result, ox-LDL could stimulate cell activity rate at a low concentration or in a short time, but it could inhibit cell activity rate at a high concentration [21–23].

As the results showed, endothelial cells' activity was inhibited by ox-LDL, while the Se-garlic oil could oppose the inhibition effect. This suggested that Se-garlic could counteract AS to a certain extent. The survey also found that the cell viability rate of each group decreased with time and this may be because ox-LDL's inhibition of endothelial cells activity was time dependent. The cell activity rate of each group first increased and then decreased, although there were not any significantly statistical differences. The reason for this needs further study, but one possible reason is that the environment for the growth of cells was changed by the mixture of the Se-garlic oil and ox-LDL. As a result of limited time and budget, we only studied Se-garlic's effect on endothelial cells' apoptosis which was stimulated by ox-LDL that was observed at a single concentration and time, but Se-garlic could effectively inhibit the effect of ox-LDL, which suggested that Se-garlic could inhibit AS.

To sum up, according to the preliminary experiments, the Se-garlic oil could inhibit ox-LDL's toxic effect on endothelial cells, and it overcomes the defect of simply mixing inorganic Se with garlic oil which will lower their bioactivity. Further studies are needed to probe into the mechanism and whether an addictive effect or synergistic effect of allicin and Se exists in Se-garlic.

## 5. Conclusion 

Endothelial cells' activity was inhibited by ox-LDL, while the Se-garlic oil could oppose the inhibition effect. This suggested that Se-garlic could counteract AS to a certain extent.
