# Corrigendum to “Effect of Selenium-Enriched Garlic Oil against Cytotoxicity Induced by OX-LDL in Endothelial Cells”

**DOI:** 10.1155/2020/8430291

**Published:** 2020-06-23

**Authors:** Evidence-Based Complementary and Alternative Medicine

In the article titled “Effect of Selenium-Enriched Garlic Oil against Cytotoxicity Induced by OX-LDL in Endothelial Cells” [1], the ECV-304 cell line has been found to be cross-contaminated by T-24, which is a bladder carcinoma cell line and not of human umbilical vein endothelial cell (HUVEC) origin [2, 3]. Hence, it is not an appropriate cell line to study endothelial cell biology.

The authors said they asked a local hospital for HUVECs and could not find in their records whether or not the type provided was indeed ECV-304 cells.

## References

[1] Yang C., Cui K., Diao Y., Du M., Wang S. (2014). Effect of selenium-enriched garlic oil against cytotoxicity induced by OX-LDL in endothelial cells. *Evidence-Based Complementary and Alternative Medicine*.

[2] ICLAC Register of Misidentified Cell Lines, http://iclac.org/databases/cross-contaminations/

[3] Capes-Davis A., Theodosopoulos G., Atkin I. (2010). Check your cultures! A list of cross-contaminated or misidentified cell lines. *International Journal of Cancer*.

